# Effect of supercritical carbon dioxide on the enzymatic production of biodiesel from waste animal fat using immobilized *Candida antarctica* lipase B variant

**DOI:** 10.1186/s12896-017-0390-1

**Published:** 2017-09-09

**Authors:** Aldricho Alpha Pollardo, Hong-shik Lee, Dohoon Lee, Sangyong Kim, Jaehoon Kim

**Affiliations:** 1 Green Materials & Process Group, Korea Institute of Industrial Technology, 89 Yangdaegiro-gil, Ipjang-myeon, Seobuk-gu, Cheonan-si, Chungcheongnam-do 31056 South Korea; 2 SKKU Advanced Institute of Nanotechnology, Sungkyunkwan University, 2066 Seobu-ro, Jangan-gu, Suwon-si, Gyeonggi-do 16419 South Korea; 3 Green Process and System Engineering, University of Science and Technology, 217 Gajeong-ro, Yuseong-gu, Daejeon, 34113 South Korea

**Keywords:** Biodiesel, Fatty acid methyl ester, *Candida antarctica* lipase B, Supercritical carbon dioxide, Waste animal fat, Methanol inhibition

## Abstract

**Background:**

Waste animal fat is a promising feedstock to replace vegetable oil that widely used in commercial biodiesel process, however the high content of free fatty acid in waste fat makes it unfeasible to be processed with commercial base-catalytic process. Enzymatic process is preferable to convert waste fat into biodiesel since enzyme can catalyze both esterification of free fatty acid and transesterification of triglyceride. However, enzymatic reaction still has some drawbacks such as lower reaction rates than base-catalyzed transesterification and the limitation of reactant concentration due to the enzyme inhibition of methanol. Supercritical CO_2_ is a promising reaction media for enzyme-catalyzed transesterification to overcome those drawbacks.

**Result:**

The transesterification of waste animal fat was carried out in supercritical CO_2_ with varied concentration of feedstock and methanol in CO_2_. The CO_2_ to feedstock mass ratio of 10:1 showed the highest yield compared to other ratios, and the highest FAME yield obtained from waste animal fat was 78%. The methanol concentration effect was also observed with variation 12%, 14%, and 16% of methanol to feedstock ratio. The best yield was 87% obtained at the CO_2_ to feedstock ratio of 10: 1 and at the methanol to feedstock ratio of 14% after 6 h of reaction.

**Conclusion:**

Enzymatic transesterification to produce biodiesel from waste animal fat in supercritical fluid media is a potential method for commercialization since it could enhance enzyme activity due to supercritical fluid properties to remove mass transfer limitation. The high yield of FAME when using high mass ratio of CO_2_ to oil showed that supercritical CO_2_ could increase the reaction and mass transfer rate while reducing methanol toxicity to enzyme activity. The increase of methanol concentration also increased the FAME yield because it might shift the reaction equilibrium to FAME production. This finding describes that the application of supercritical CO_2_ in the enzymatic reaction enables the application of simple process such as a packed-bed reactor.

## Background

There are a variety of feedstocks that can be used for biodiesel production such as vegetable oil, animal fat, algal lipid and waste oil and so on. Currently, the commercial biodiesel is mainly produced from vegetable oil, however, it is also needed for human consumption. Because of consumers’ necessity, the price of such oil makes the overall production cost of biodiesel less competitive than the conventional petroleum fuel. Therefore, there is a need in finding a new type of feedstock with lower cost and less conflict in food production. Waste animal fat is one of promising feedstocks to be utilized for biodiesel since it is usually disposed instead of human consumption and requires cost for its disposal management [1]. The availability of waste animal fat is also high especially in countries with high consumption of meats such as the U.S. and South Korea [2]. Despite of these beneficial properties, waste animal fat contains higher percentage of free fatty acid than the edible oil [3].

The most common method to obtain biodiesel is the transesterification of triglycerides with the presence of base-catalyst and acyl acceptor such as short chain alcohol and alkyl acetate. The commercial biodiesel process uses a homogeneous base catalyst which quantity escalates along the production capacity of biodiesel. As a result, the separation of product from catalyst is inevitable and a huge amount of basic waste is produced in biodiesel industry resulting in environmental management issues. In such process, the raw material with high content of triglyceride is necessary. If the raw material contains a considerable amount of free fatty acid and water, it might cause several issues, that is, the saponification between base catalyst and free fatty acid, the decrease of catalytic activity because of dissolution of catalyst into the water, and increasing the workload of product purification step. As the consequence, for the utilization of waste fat with commercial method, the addition of slow-rate acid-catalysed esterification step is needed to remove the free fatty acid before the base-catalysed process [4]. This means that the waste animal fat processing not only increase the cost of separation process of both raw material and product, but also the expense of waste treatment process.

The difficulty of waste animal fat utilization using alkaline catalyst can be overcome by deploying enzymatic process. Lipase is known to convert both free fatty acids and triglycerides into biodiesel with the presence of acyl-acceptor but the conversion rate is slow due to the diffusion disturbance caused by its byproduct and alcohol poisoning to enzyme [5]. Furthermore, the crude enzyme stability is low and the price to obtain high-purity enzyme is expensive so that the commercialization of enzymatic biodiesel is still unfeasible to be executed. Some studies initiated the use of solvent and co-solvent to reduce the negative effect of methanol and glycerol and showed promising results. Su and Wei reported that 61% of conversion was obtained from the transesterification of *Jatropha curcas* L. seed oil on Novozym 435 using the co-solvent system consisting of t-pentanol and isooctane [6]. Soumanou and Bornscheuer discovered that the addition of n-hexane on the transesterification of sunflower oil on various lipases resulted in higher than 70% of yield. [7]

Supercritical carbon dioxide (scCO_2_) recently attracted attentions to be utilized as solvent for biodiesel enzymatic transesterification. This is due to the high diffusivity provided in supercritical fluid environment which enables enzymes to interact faster with the reactants [8]. Other advantage is that, the scCO_2_ is not consumed in the transesterification and can be separated instantly from the product because simple depressurization can easily turn scCO_2_ phase into gas. Several studies have reported the use of scCO_2_ or near-critical CO_2_ for lipase-catalysed transesterification of oil with stepwise addition of methanol into the reaction system and the end result is achieved faster than doing the same reaction in atmospheric condition [9–11]. Reaction parameters such as pressure, temperature, exposure time and depressurization rate on lipase activity in supercritical fluid have also been observed [12]. Most studies on the enzymatic reaction have applied the stepwise methanol addition to minimize the methanol concentration in reaction mixture. However, in the view of reaction engineering, low methanol concentration causes low reaction rate and makes the required reactor volume larger. The purpose of this study was to investigate the biodiesel production from waste animal fat using novel enzyme for the commercial application. Especially, the effect of scCO_2_ and methanol concentration on the inhibition to lipase activity was focused on.

## Methods

### Chemicals

The *Candida antarctica* lipase B variant (CalB 1422) immobilized on a methacrylic resin supplied from Korea Research Institute of Bioscience and Biotechnology (KRIBB) was used. It was obtained by directional evolution of wild-type CALB (Candida antartica lipase B), and *Saccharomyces cerevisiae*, which secretes it, was fermented to produce an enzyme. The enzyme was concentrated by diafiltration and immobilized on Lewatit VP OC 1600 resin. The immobilized concentration was 25–30 mg/g resin. The feedstocks used in this study were soybean oil (SBO), crude waste animal fat (CAF), and refined waste animal fat (RAF). The commercial SBO (CJ CheilJedang, Korea) was purchased from local grocery, and CAF and RAF were supplied by Taegok Oils & Fats. The RWF was the CAF pretreated with filtration and pressing. Liquefied carbon dioxide (99.999%, Dae Myung Specialty Gas, Korea), methanol (99.9%, Sigma-Aldrich, USA), methyl nonadecanoate (98.0%, Sigma-Aldrich, USA) and toluene (99.9%, Sigma-Aldrich, USA) were purchased and used without further purification.

### Transesterification at ambient pressure

The transesterification of SBO, CAF and RAF at ambient pressure was carried out in a 100 mL glass bottle. 2 g of immobilized CalB 1422 and 20 g of feedstock were put into the glass bottle. To initiate the reaction, 2.4 g of methanol was added by different two ways, i. e., by batch type (B method, added once at the beginning of the reaction) and semi-batch type (SB method, added 6 times stepwise by 0.4 g every hour). The reaction system was conditioned in 40 °C inside a shaking water bath with shaking speed of 200 rpm for 6 h.

### Transesterification in supercritical CO_2_

**Fig. 1:**
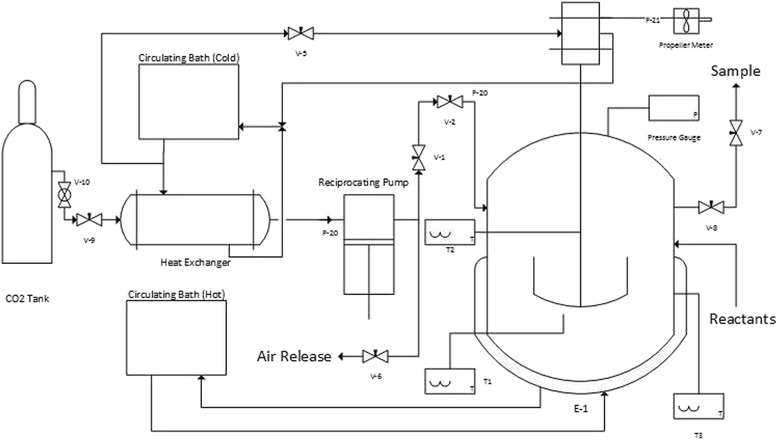
Schematic diagram of high-pressure reactor for the enzymatic transesterification in supercritical CO_2_

The transesterification of feedstock in supercritical CO_2_ was studied using a high-pressure batch reactor with 140 mL in volume. The schematic diagram of the high-pressure reactor was shown in Fig. 1. 5 g of enzyme resin, 50 g of feedstock and 6 g of methanol was put into the reactor in order. Then, the reactor was assembled so that the inside part was isolated from the environment. After finishing the assembly, liquefied CO_2_ was introduced into the reactor to the predetermined pressure by a high-pressure pump (Hanyang Accuracy, HKS-1000). The pressure was controlled by the amount of introduced CO_2_, and the temperature was controlled using a circulating water bath (Jeio Tech, W100495). Reaction was started when the agitator was turned on. The reaction last for 6 h with taking sample every 1 h. The whole procedure is repeated for 25 g and 10 g of feedstock mass. With the same procedure above, the 10 g feedstock mass is used in the reaction system with variation of methanol concentrations. The concentrations (in gram methanol/g feedstock) used during the experiments were 12%, 14%, and 16% based on feedstock mass.

### Analytical method

Analysis of fatty acid methyl ester (FAME) yield was carried out based on EN14103–2011 method. Approximately fifty millilitres of samples was weighed in a 5 mL vial then 5 mL of toluene was put inside the vial along with 50 mg of internal standard (methyl nonadecanoate). Biodiesel yield was obtained by measuring composition of FAME in the samples using gas chromatography (Agilent 6890) with a capillary column (HP-INNOWax, 30 m/0.320 mm/0.5 μm). Afterwards, the FAME yield was calculated based on the peak areas obtained from chromatogram using the following equation:

$$ C=\frac{\varSigma A}{A_{ISTD}}\times \frac{C_{ISTD}\times {V}_{ISTD}}{M_{sample}} $$

Where:

- C = FAME yield in mass fraction
- *A*_*ISTD*_ = Peak area of internal standard
- ΣA = Total peak area of FAME without A_ISTD_
- C_ISTD_ = Concentration of ISTD in toluene
- V_ISTD_ = Volume of ISTD used in the analysis
- M_sample_ = Mass of sample used in the analysis

All experiments were at triplicated to confirm the repeatability and the average values were provided.

## Results

**Fig. 2:**
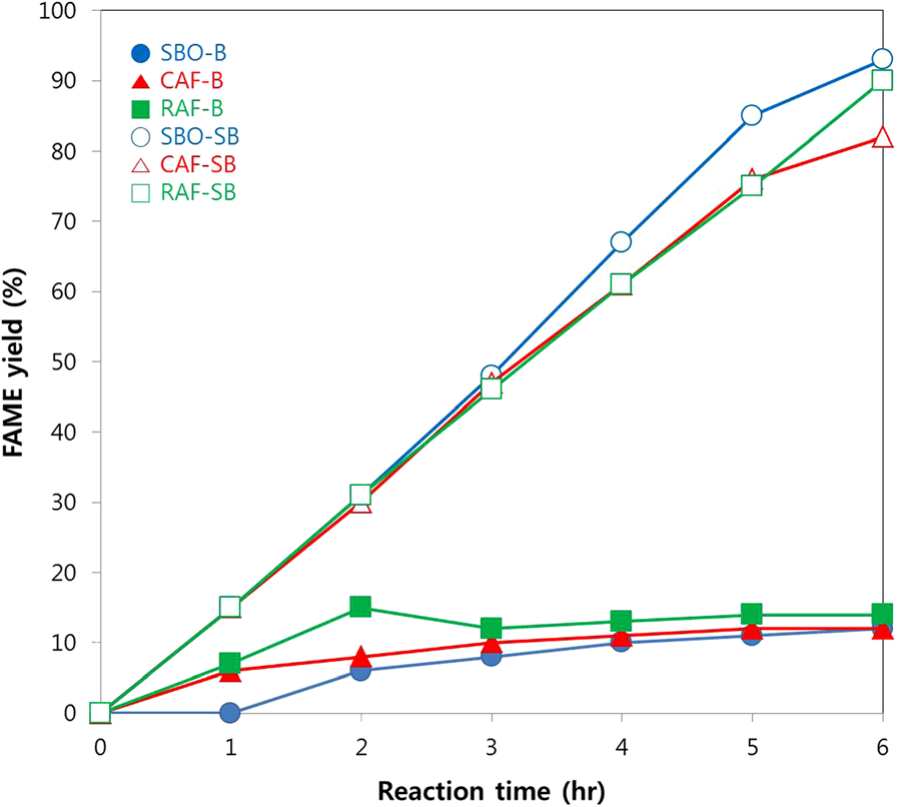
Change of FAME yield from enzymatic reaction of different feedstocks under atmospheric pressure for 6 h of reaction at 40 °C and 200 rpm mixing in shaking bath. Closed symbols and open symbols are corresponded with the results obtained using B method and SB method respectively

To investigate the activity of CalB 1422 on different feedstock, a series of experiments were carried out in an atmospheric condition. The FAME yields from SBO, CAF and RAF were shown in Fig. 2. In case of the B method, the FAME yield from SBO, CAF and RAF reached at 12%, 12%, and 14% after 6 h respectively. However, in case of the SB method at the same conditions, the FAME yield from SBO, CAF and RAF increased to 93%, 82%, and 90% respectively.

**Fig. 3:**
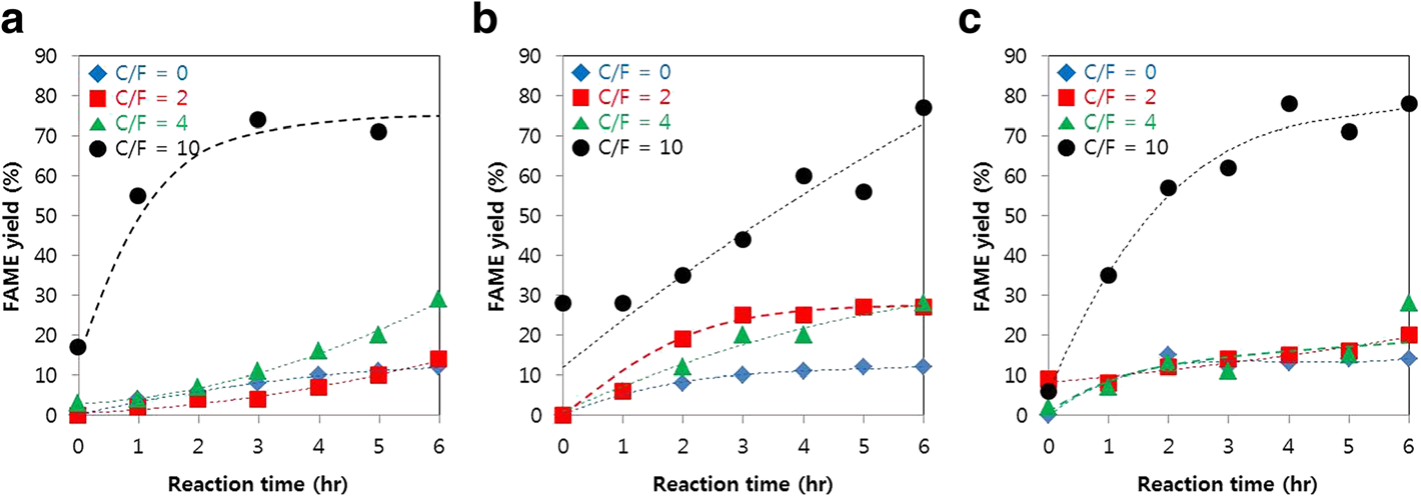
FAME yield profile from the enzymatic reaction of **a** SBO, **b** CAF, and **c** RAF in different ratios of supercritical CO_2_

The enzymatic reaction in supercritical CO_2_ was done for 6 h at 40 °C and pressure maintained above its supercritical condition of around 150 bar. The CO_2_ fed into the reactor is approximately 100 g. To observe the effect of CO_2_ to feedstock ratio (C/F), the amount of feedstock (SBO, CAF, RAF) and methanol was changed at the fixed amount of CO_2_ fed. The FAME yields from the C/F of 2:1, 4:1, 10:1 were shown in Fig. 3. When utilizing SBO as raw material, the FAME yield reached 14%, 29%, and 81% after 6 h for the C/F of 2:1, 4:1 and 10:1 respectively. The CAF conversion showed FAME yield of 27%, 28%, 77% for the C/F of 2: 1, 4: 1, and 10: 1 respectively. The RAF, which is the main raw materials for this research, showed FAME yield of 20%, 28%, 78% for the C/F of 2: 1. 4: 1, and 10: 1 respectively.

**Fig. 4:**
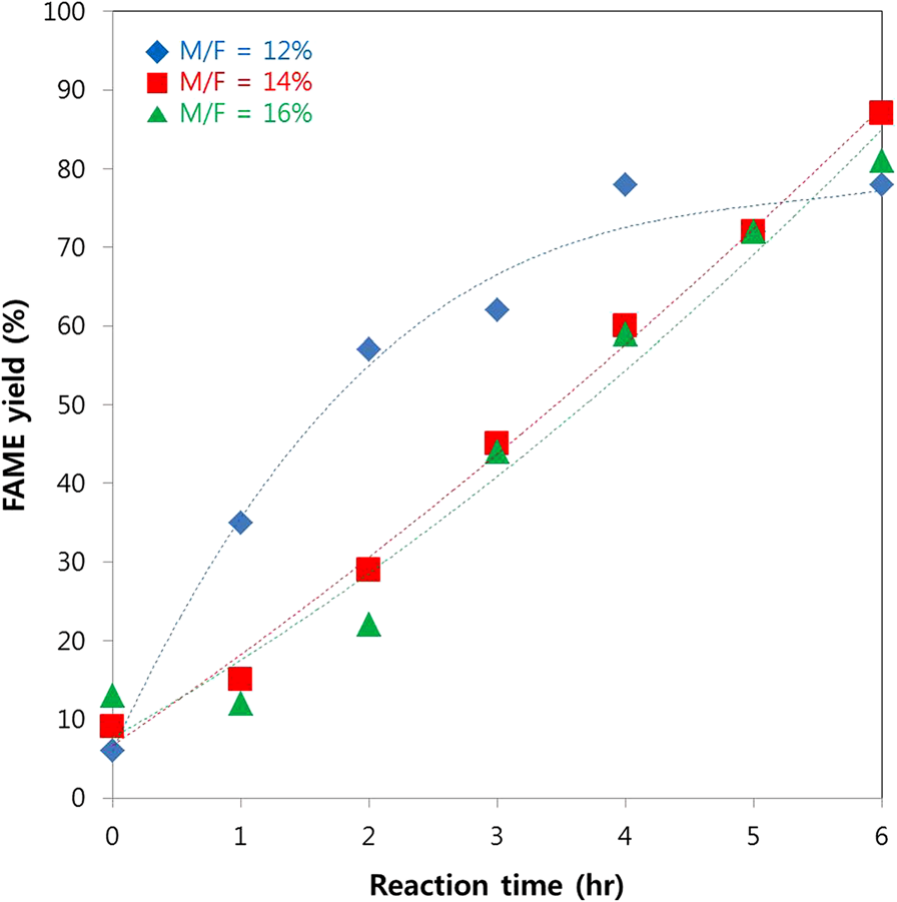
FAME yield profile of enzymatic transesterification of RAF in scCO_2_ with varied M/F ratio. The reaction temperature and pressure was 40 °C and 150 bar, and the C/F ratio was 10:1

To obtain the higher reaction rate, higher methanol concentrations were tested at the C/F of 10:1. The results from 12%, 14% and 16% of methanol/feedstock ratio (M/F) were shown in Fig. 4. It was found that FAME yields were 81% and 87% in 6 h when the M/F was increased to 14% and 16% respectively. This result is higher than stoichiometric methanol concentration which reached around 77.73% conversion.

**Fig. 5:**
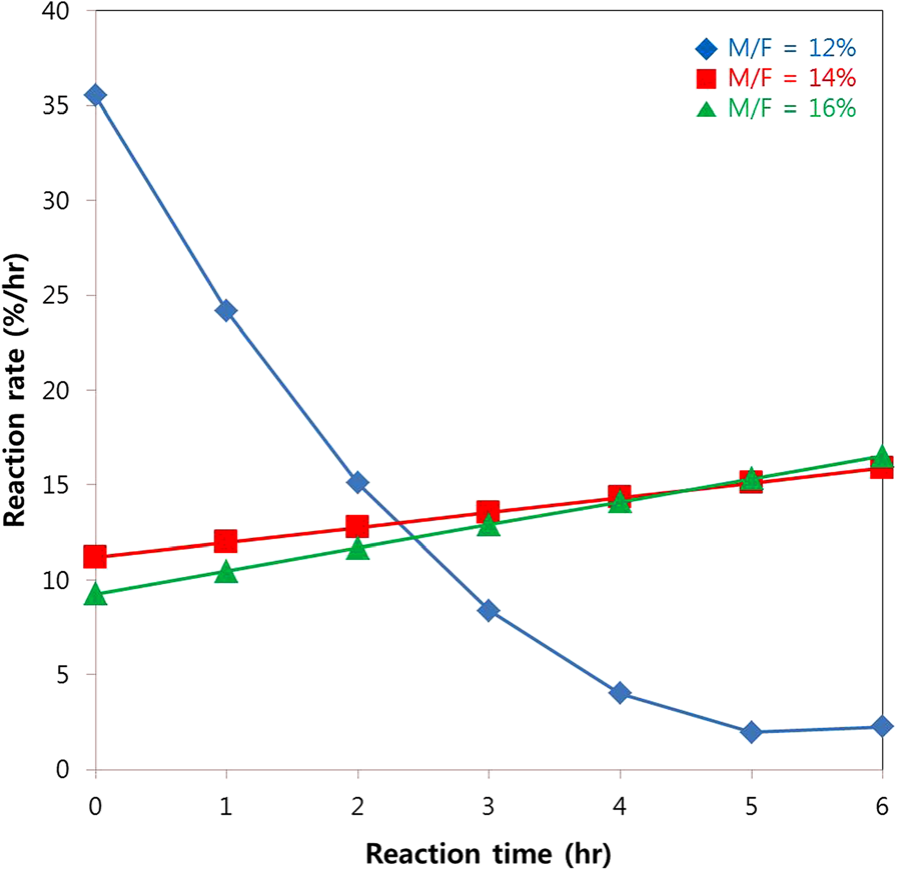
Reaction rates derived from the findings in Fig. 4

The reaction rates derived from the slopes of curves in Fig. 4 were shown in Fig. 5. At the M/F ratio of 12%, the relatively high initial rate and the prominent decrease of reaction rate over time were observed. At the M/F ratio of 14% and 16%, the initial rate was relatively low while the reaction rate increased slightly along with reaction time.

## Discussion

In the B method at atmospheric pressure, the conversion of feedstock to biodiesel was higher than 80% in all feedstocks, therefore, it was confirmed that the CalB 1422 used in this study has good performance even in atmospheric condition. The result of enzymatic transesterification in the B method in atmospheric condition showed lower conversion of feedstock into biodiesel compared to the SB method. These results are due to methanol inhibition to the enzyme and in agreement with previous reports [5]. The trend in all types of feedstock in the B and SB method, however, showed the same trend of biodiesel yield over time. This proved that the enzyme activity was not affected by the types of fatty acid present in the feedstock.

The FAME yields from CAF and RAF in supercritical CO_2_ were higher than those from SBO when the C/F ratio was 2: 1. This might occurred because the melting point of animal fat is higher than that of SBO due to more saturated fatty acids. When too much animal fat was dispersed in scCO_2_, homogeneous state was not formed. Instead, the saturated fatty acid contributed to solidification of fat in the reaction system. This resulted in fat covering the surface of enzyme while the fat was being transesterified at the same time. The fat covering over the enzyme surface might lower the methanol poisoning to the enzyme active sites. However, when the concentration of feedstock was lowered to C/F ratio of 4: 1 and 10: 1, the FAME yield showed the same trend for all types of feedstock. This result suggested that at a certain supercritical CO_2_ concentration, the fat or oil can form homogeneous mixture with the scCO_2_. In all types of feedstock, the FAME yield generally increased with increasing the C/F ratio. The dramatic increase of the reaction rate in the C/F ratio of 10: 1 showed that the oil was dissolved completely in scCO_2_ in that ratio. These observations can be explained with the following two aspects. First, the enhancement of mass transfer between reactants and enzyme by supercritical CO_2_ can accelerate the overall reaction rate, and this effect becomes more significant when more reactants are dissolved in scCO_2_. However, the addition of CO_2_ can spontaneously have adverse effect because the dilution of reactants concentration makes the intrinsic reaction late low. The second issue is related to the methanol inhibition on the enzyme. While the dilution lowered the reaction rate because of lower oil concentration, it also lowered the methanol concentration in the system. Low methanol concentration might lower the methanol interaction with the enzyme and reduced the enzyme deactivation which further prevents the decrease of reaction rate due to enzyme deactivation. In conclusion, it is considered that, despite of the dilution reaction rate reduction, the supercritical fluid removed the limitation of mass transfer and the deactivation of enzyme to result in increasing reaction rate.

The higher methanol concentration might contribute in shifting the reaction equilibrium towards the production of biodiesel. Therefore, higher FAME yield was observed along the increase of methanol concentration. However, if the methanol concentration is too high, it might inhibit the activity of enzyme and in turn decrease the product yield. Overall, the FAME yield is increased along with the increase of methanol concentrations as can be seen in Fig. 4. In the 12% methanol experiment, higher initial rates was observed (see Fig. 5) than the other two variants but the rates decreased significantly starting from the fourth hours. The decrease probably caused by the decrease of methanol as substrates which resembles Michaelis-Menten kinetics trend. Furthermore, this case proved that the reaction rate is increased due to removal of mass transfer limitation and the supercritical fluid CO_2_ could reduce the methanol inhibition. However, the decrease of reaction rate was insignificant when the methanol concentrations were 14% and 16%. This indicates that the reaction can still be continued even further although the initial rates of reaction became low due to methanol poisoning to enzyme. The stable profile of reaction rates in 14% and 16% methanol experiment indicates that there is a balance between methanol inhibition rate and transesterification rate.

## Conclusion

In this paper, the role of supercritical CO_2_ on the enzymatic transesterification of waste animal fat was investigated. It was confirmed that at a high CO_2_ to feedstock ratio, the supercritical CO_2_ had the effect to lower the interaction between methanol and the enzyme and further reduce the methanol intoxication to enzyme. More than 77% of FAME yield was achieved in 6 h of reaction time using the total addition method. Increasing methanol concentration reduced the initial reaction rate while it increases the final FAME yield. Based on these findings, it is concluded that scCO_2_ process can be applied to the packed-bed reactor which requires small reactor volume and high reactant concentration at the inlet of reactor. Therefore, the packed-bed reactor is expected to be available for the research on the continuous reaction of the enzymatic transesterification and scale-up study, which are to be further studied.

## Abbreviations

B method: Batch method
C/F: CO_2_ to feedstock ratio
CAF: Crude waste animal fat
CalB 1422: *Candida antarctica* lipase B variant
FAME: Fatty acid methyl ester
M/F: Methanol to feedstock ratio
RAF: Refined waste animal fat
SB method: Semi-batch method
SBO: Soybean oil
scCO_2_: Supercritical carbon dioxide
